# Visceral Origin: An Underestimated Source of Neck Pain. A Systematic Scoping Review

**DOI:** 10.3390/diagnostics9040186

**Published:** 2019-11-12

**Authors:** Ángel Oliva-Pascual-Vaca, Carlos González-González, Jesús Oliva-Pascual-Vaca, Fernando Piña-Pozo, Alejandro Ferragut-Garcías, Juan Carlos Fernández-Domínguez, Alberto Marcos Heredia-Rizo

**Affiliations:** 1 Department of Physiotherapy, Faculty of Nursing, Physiotherapy and Podiatry, University of Sevilla, 41009 Sevilla, Spain; angeloliva@us.es (Á.O.-P.-V.); gonzalez.salle@gmail.com (C.G.-G.); amheredia@us.es (A.M.H.-R.); 2 Escuela de Osteopatía de Madrid, 28002 Madrid, Spain; 3 Department of Physiotherapy, Universitary School of Osuna, University of Sevilla, 41640 Sevilla, Spain; ferppozo@hotmail.com; 4 Department of Nursing and Physiotherapy, University of the Balearic Islands, 07112 Palma de Mallorca, Spain; alejandro.ferragut@uib.es (A.F.-G.); jcarlos.fernandez@uib.es (J.C.F.-D.)

**Keywords:** referred pain, visceral pain, diagnosis, phrenic nerve, neck pain

## Abstract

The diagnosis of neck pain is challenging. Many visceral disorders are known to cause it, and clinical practice guidelines recommend to rule them out during neck pain diagnosis. However, the absence of suspicion of any cause impedes one from establishing that specific aetiology as the final diagnosis. To investigate the degree of consideration given to visceral aetiology, a systematic search of trials about neck pain was carried out to evaluate their selection criteria. The search yielded 309 eligible articles, which were screened by two independent reviewers. The PEDro scale score was used to assess the methodological quality of the studies. The following information was retrieved: number of authors affiliated to a clinical or non-clinical institution, number of citations in the Web of Science, study aims, characteristics of participants, and eligibility criteria. The top 15 most cited trials, and the 15 most recent studies about treatment efficacy in neck pain, published in first quartile journals of the Journal Citation Reports, were selected. Females represented 67.5% of participants. A single study was of poor methodological quality (4/10). Based on the eligibility criteria of the articles that were systematically reviewed, it would appear that visceral aetiology was not considered in eighty percent of the trials on neck pain, showing a low level of suspicion both in research and clinical settings.

## 1. Introduction

Neck pain (NP) constitutes a major health problem. Its prevalence varies from 4.8% to 79.5%, and is more common in females and in high-incomes countries [1]. It is ranked the 4th most disabling condition as measured by years lived with disability [2]; hence, it poses a substantial economic burden due to extended periods of sick leave and high use of health services [3]. Those individuals with a precise pathoanatomical cause for their NP, e.g., radiculopathy [4,5], facet joint pain [6], chronic rheumatic diseases [7], or cancer, are categorized as having specific NP. Yet, patients without a well identified source for their NP are labelled as having idiopathic, mechanical, or non-specific NP, which is the most common type [8].

As a sign of visceral suffering, pain originating in internal organs is amongst the most frequent forms of pain experienced by individuals in the course of life, and pain involving internal organs is a major occurrence in the clinical setting [9,10]. The rule in visceral nociception is that pain is referred to somatic tissues, being felt at a site other than the affected viscera [10]. Visceral referred pain (VRP) occurs, as secondary hyperalgesia, in somatic areas neuromerically connected with the affected organs [10]. The overlap of somatic and visceral afferent information into a shared neural pathway seems to be related to a misinterpretation at peripheral, spinal, or supraspinal levels [10,11]. The precise substrate underlying this phenomenon remains unknown [12]; it has been stated that it might explain the strong association between back pain and digestive disorders [13].

**Figure 1:**
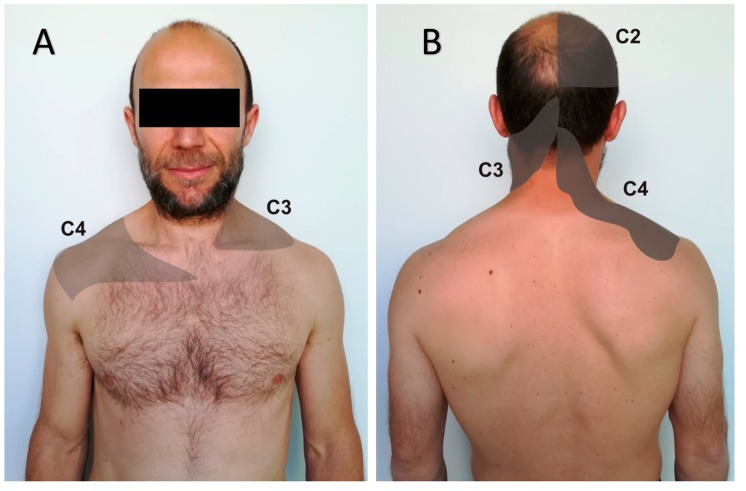
Pattern of visceral referred pain along the C2–C3–C4 dermatomes. (**A**) Anterior view; (**B**) posterior view.

Understanding and awareness of referred pain is key for a precise diagnosis of the pain source [14]. Previous evidence shows that gastrointestinal, biliary, renal, hepatic, heart, and pulmonary disorders may evoke referred pain to the upper quadrant of the body, including the neck region [15]. The discrimination between visceral and somatic sources of pain is not always evident, and if it is not achieved, it may lead to extensive diagnostic procedures and ineffective treatment approaches [16]. Visceral disorders may evoke referred altered sensitivity, e.g., hyperalgesia or allodynia [17]. For instance, the radiation of pain to the neck and/or upper extremity that occurs during acute coronary syndromes [18] is experienced in more than 65% of cases [19]. Eighty-eight percent of patients with colonoscopy-induced splenic injury complain of pain along the C3–C4 dermatomes due to irritation of the diaphragm or distention of the splenic capsule [20] (Figure 1) [21]. That happens during attempts at sheath insertion into the right or middle hepatic vein in liver biopsy as well [22]. Further, it can also be caused by more common, frequently long-lasting, and not so life-threatening conditions, such as hiatal hernias and gastroesophageal reflux disease [23].

When routinely evaluating patients with NP, it is easy to miss manifestations of an underlying disease, and misdiagnose neck disorders of visceral origin [15,24]. Clinical practice guidelines for the management of NP recommend a detailed physical examination to rule out the possibility of VRP in individuals with NP [25]. Hence, clinical trials assessing treatment efficacy in NP should exclude participants with suspected VRP after a comprehensive evaluation. Otherwise, this selection bias would show an underconsideration of that source of NP, and in addition, result in a likely incorrect estimation of the treatment’s effect size. Therefore, the aim of the systematic review was to investigate to what extent the top 15 most cited and the 15 most recent clinical trials published in high impact journals, by November 2018, that assessed treatment outcomes in patients with NP, took into account VRP when establishing their eligibility criteria.

## 2. Materials and Methods

The present systematic review was performed according to the Preferred Reporting Item for Systematic Reviews and Meta-Analyses extension for Scoping Reviews guidelines [26]. It has been registered in the International Prospective Register of Systematic Reviews (PROSPERO), with registration number CRD42018101987.

### 2.1. Data Sources and Search Strategy

One author (C.G.-G.) conducted a systematic computerized search between November and December 2018 using the Web of Science database. The search used the key terms neck pain and trial, and considered the following limitations: both key terms being included in the title of the article; language—English/Spanish/Italian/French; and having a publication date between January 1995 and November 2018.

### 2.2. Study Selection

In order to obtain the information from high-quality studies, eligible articles were the top 15 most cited clinical trials published between 1995 and 2018, and the 15 most recent studies included in high impact journals (first quartile of the Journal Citation Reports in the year of publication of the study), which assessed any therapy for subjects suffering from NP. Those articles with any of the following characteristics were excluded: NP patients with only a traumatic, surgical, or neurological origin for the condition; study protocols for clinical trials; studies including only elderly adults (older than 65 years), or including adolescents or children (younger than 18 years); or a lack of a clear description of the eligibility criteria. All relevant titles were saved in a reference manager (EndNote^®^, version X8.2, Thomson Reuters). Two researchers (C.G.-G. and Á.O.-P.-V.) independently performed the assessment of the studies in a blinded and standardized manner, taking into account the eligibility criteria previously set out. In the case of a disagreement, the issue was discussed together with a third member of the research team (A.M.H.-R.) until a final consensus was reached.

### 2.3. Assessment of the Methodological Quality

The Physiotherapy Evidence Database (PEDro) scale score was used to assess the methodological quality of the clinical trials. The PEDro scale is an 11-item tool where items are scored as either absent (0) or present (1), except for item 1 that refers to external validity of the study. A final score from 0 to 10 is given. The PEDro scale is a valid [27] and reliable [28] tool to rate the methodological quality of clinical trials. A cut-off of at least 5 or 6 points is required for a study to be of adequate quality [29]. PEDro scores were extracted from the PEDro database. Two independent raters (J.C.F.-D. and A.F.-G.) evaluated, with the PEDro scale, those trials not included in the PEDro database. A final consensus about the final score was reached, together with a third member of the research team (A.M.H.-R.), whenever necessary.

### 2.4. Data Extraction

Once the studies were selected, two authors (C.G.-G. and J.O.-P.-V.) independently retrieved the following information from each article following a standardized form: the number of authors affiliated with a clinical institution, e.g., hospital, private practice, or health-center, and the number affiliated with a non-clinical institution, e.g., a university or research center; total number of citations in the Web of Science; the PEDro scale score; aims of the study; sample size and characteristics of participants (distribution by sex, mean age and pain duration); and eligibility criteria (inclusion and exclusion criteria). Data collected from the studies we included were pooled into tables.

## 3. Results

### 3.1. Study Selection

**Figure 2:**
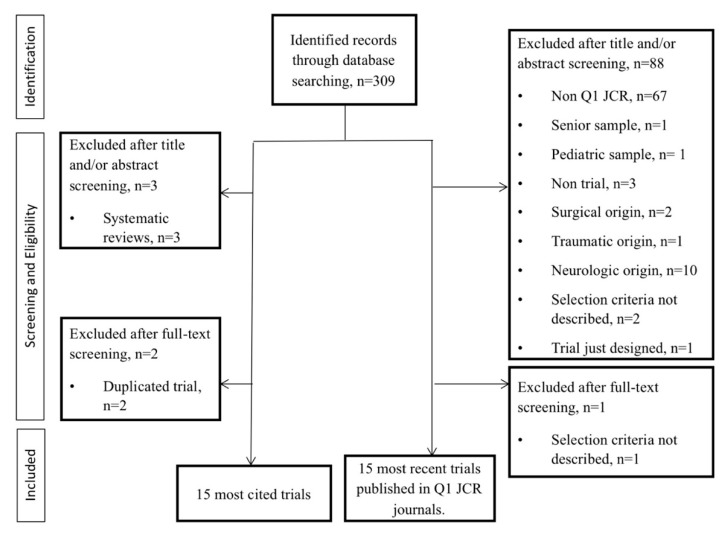
Flowchart diagram of the study selection process (Preferred Reporting Item for Systematic Reviews and Meta-Analyses, PRISMA, guidelines). Q1, first quartile; JCR, Journal Citation Reports.

The search strategy resulted in a total of 309 relevant articles that were retrieved through the Web of Science database. Then, 94 studies were excluded for not matching the eligibility criteria. From the remaining 215 articles, 30 of them were finally included. All selected studies were written in English. Two of the top 15 most cited articles were excluded and replaced by the next most cited clinical trials in the list. The reason was that the sample population and the eligibility criteria used were the same as in other studies with a higher number of citations that were published by the same research groups and that had been already included for further analysis. Figure 2 shows the flow diagram for the study selection process.

### 3.2. Study Characteristics

All of the clinical trials which were included were randomized and controlled. Detailed descriptions of articles included in this systematic review are presented in Table 1; Table 2. Researchers from non-clinical institutions authored more than 90% of the studies (28 out of 30), while authors from clinical institutions, e.g., hospitals, health-centers, and private practices, participated in 79% of trials (21 out of 30). The studies included a total of 4467 participants, with females representing 67.5% of the total (3017 females). One clinical trial did not clearly specify the sex distribution of the study sample [30]. Two studies recruited exclusively females [31,32], and only two of them selected more male than female individuals [33,34]. The mean age of participants was between 35 and 53 years, with one study including younger participants (mean age of 21 years) [35].

### 3.3. Methodological Quality of Studies

The assessment of the methodological quality by means of the PEDro scale revealed that, in general, the top 15 most cited clinical trials denoted adequate to good methodological quality, with a final score of six points or higher, except for one study that scored five out of 10 points (Table 1) [36]. Similar findings were observed amongst the 15 most recent articles published in high impact journals (Table 2), although one study denoted poor methodological quality (four out of 10) [37]. One of all studies included achieved excellent methodological quality (10 out of 10) [38]. The reliability between coders for those studies whose scores were not available in the PEDro database was almost perfect (Kappa = 0.84) [39].

### 3.4. Eligibility Criteria (Inclusion and Exclusion Criteria) Used by Trials

Of all trials analyzed, a single study [38] defined stringent inclusion criteria to avoid the recruitment of participants with possible VRP. This study included patients with a positive response to cervical facet joint nerve blockers; e.g., 80% pain relief and the ability to perform previously painful movements. With regard to the exclusion criteria, five clinical trials listed them to avoid the selection of individuals with suspected VRP as the cause of their NP. Two studies explicitly excluded participants with “NP referred from peripheral joints or viscera” [47,50]. The other three studies excluded individuals who suffered from NP with a “non-mechanical cause” [45], reported “any medical sign suggestive of a non-musculoskeletal aetiology” for their NP [43], or were diagnosed with a “specific cause for the neck pain”; e.g., organic disorders or systemic diseases [48]. A “clear aetiology” [57] or a “specific cause” [57,58] for the NP was also listed as an exclusion criterion in three other trials. Yet, none of the latter studies mentioned visceral or organic disorders as possible specific causes for the NP. Some other visceral sources of NP were enumerated in other clinical trials: (a) hepatitis [37]; (b) systemic disorders, including metabolic disease [30,35,36,41,46,57,58]; (c) abuse of alcohol and drugs [37,54]; (d) rheumatic disease [31,32,33,35,37,46,49,51,52,53,55,56,58]; (e) cancer [30,31,35,37,42,51,52,53,55,56,57]; (f) HIV [37]; and (g) infection [30,33,34,40,45,47,57]. Pregnancy was also included as exclusion criteria in almost half of the trials [30,32,34,37,38,40,46,50,51,52,54,55,56,59]. Two clinical trials did not refer to any possible visceral aetiology of neck pain in their inclusion and exclusion criteria [44,60].

## 4. Discussion

The present findings suggest that amongst the most cited and the most recently published clinical trials assessing treatment efficacy in NP there is a lack of consideration for VRP as a plausible source of NP. This appears to be the case when the eligibility criteria for recruiting participants are analyzed. Only 20% of all selected trials (six out of 30) defined stringent enough criteria to avoid the recruitment of individuals with a suspected visceral referred NP. All these studies were amongst the top 15 most cited articles. Three other trials excluded patients with a specific aetiology or cause for their NP, although authors did not even mention the visceral area. This implies that most of the assessed trials might have included patients with a visceral source of NP despite the fact that the visceral disorder would not be the target of the treatment. Therefore, it might show an underconsideration of this neck pain aetiology; in addition, incorrect estimations of the effects or efficacies of the interventions could have occurred.

### 4.1. Eligibility Criteria Used by Trials to Select NP Patients

Manchikanti et al. [38] considered as eligible, those patients with a positive response to cervical facet joint nerve blockers, which excluded individuals with visceral referred NP. The rest of the clinical trials, however, established general inclusion criteria; e.g., neck stiffness; mechanical pain with reproducibility of symptoms during physical examination, neck movement, or posture maintenance; and myofascial pain syndrome, among others. All these symptoms mainly refer to increased local sensitization and muscle tension, which can be due to a visceral issue. A primary visceral disorder may also be accompanied by hyperalgesia of the painful area, and is often associated with sustained muscle contraction [61], and it may extend to subcutaneous tissues when the visceral disorder is persistent [62]. Furthermore, the increased muscle tone may explain mechanical symptoms and lead, in the long-term, to the presence of myofascial trigger points and myofascial pain syndrome [63,64]. Hence, visceral pain can evoke many different neck symptoms, including muscle spasms in addition to pain [15], and when sustained, may help to develop central sensitization and cortical changes [61]. Despite all this, surprisingly, the visceral aetiology of NP was only properly considered in six clinical trials [38,43,45,47,48,50]. These findings may imply a general misdiagnosis of NP in research and clinical scopes, thus patients with visceral referred NP might not receive the most accurate therapeutic approach. Visceral pain shares many features with pain from deep somatic structures and requires well-developed propedeutics to avoid inadequate diagnosis and treatment [65]. The test for cutaneous allodynia appears to have the greatest likelihood of identifying a visceral source of pain compared to somatic sources of pain [16]. Therefore, a detailed clinical history, physical examination, and supplemental laboratory and imaging examination is needed to diagnose the primary source of pain [61,64], because the somatic manifestation will persist until the visceral disorder resolves by itself or has been discovered and treated [23,66]. This discovery is more likely to happen in subjects with constant neck pain and/or aggravation of visceral symptoms, but is less likely in recurrent, episodic NP and/or subtle visceral symptoms [15]. Additionally, in cases of chronic NP, the right diagnosis can be favored along the course of the disease by the obtaining of abnormalities in blood, urine, digestive, heart… tests, either developed ad hoc to diagnose the origin of NP or during medical assessments for other reasons. Since many of the visceral disorders which may trigger NP are chronic, they enable the presence of either episodic (due to episodic aggravation of the visceral disorder), recurrent (due to frequent aggravation of the visceral disorder), or chronic NP.

### 4.2. Somatic Consequences of Visceral Disorders

Visceral referred NP is linked to the involvement of the vagus and/or phrenic nerves. The nociceptive input from any of the organs innervated by the vagus nerve sensitizes the trigeminocervical nuclear complex that descends to C3 or C4 levels, and may trigger a headache [67] and/or NP [14]. The phrenic nerve is a motor and sensitive nerve formed by C3–C4 roots, with C5 as an accessory root. Either directly or through celiac connections, it supplies the diaphragm, pleura, right atrium [68] pericardium [68,69], esophagus [70], peritoneum [68,71], stomach [15], falciform and coronary ligaments of the liver [72], the Glisson capsule [72,73], the hepatic vein [74], the inferior vena cava [68,70,74], the liver [68,72,75] (parenchyma) [74], the gallbladder [72,76,77] and the rest of the biliary tract [71,72,77]—including the duodenal papilla and the sphincter of Oddi [77,78] —, the pancreas [15], the small intestine [15], and the suprarenal glands [68,70,71]. Hence, disorders of many of these structures, such as the pancreas, or even the spleen or kidneys, can evoke referred pain along the C3–C4 dermatomes either due to the autonomic connections, diaphragmatic pressure, or peritoneal irritation [15]. This has been described as “phrenic pain” [23].

To date, there is no data about the prevalence of NP of visceral origin in general practice or musculoskeletal settings. Nevertheless, in a previous study [79] that seemed not to consider the visceral aetiology, a well identified cause of the NP was not found in 32% of patients receiving a complete evaluation in a private pain clinic, where, probably, those NP patients with the most severe symptoms are a majority. As well, a history of previous trauma was present in most of the patients. However, the authors do not clarify if the inability to achieve a specific cause was more frequent in patients who had previous trauma or in those who did not.

It is important to consider the prevalence of the causes of any condition, because that determines the pretest probability, the order of the investigations, and it can also affect to prognosis. The prevalence of visceral disorders that may trigger pain in the neck-shoulder area is high. It is estimated that the one-year prevalence of gastroesophageal reflux with weekly symptoms is 14% [80], and 15% of Americans have silent gallstones, 10–18% of whom develop biliary pain [81]. Besides, non-alcoholic fatty liver is present in around 30% of the population in western countries [82]. Females are more prone than males to have widespread hyperalgesia from recurrent visceral pain [61], and also NP is more prevalent in females. Further, NP of high intensity/low disability or high disability is strongly related to cardiovascular and digestive disorders [83]. That may suggest, eventually, a visceral origin for the pain, which together with cervical spasms have been observed in animal models [84] and in humans [23] with gastric or esophageal disorders. It is also known that NP is highly associated with obesity [85], LDL cholesterol [86], and metabolic syndrome [87]. For instance, the prevalence of NP in those with metabolic syndrome ranges between 16% for males and 25% for females [87]. This is remarkably important because fatty liver, obesity, and metabolic syndromes entail hepatic suffering; e.g., increased pressure, swelling, and hepatomegaly. Phrenic afferents in the hepatic parenchyma, hepatic veins, and the inferior vena cava just need light pressure to respond [74,88]. Further, all this can stretch and sensitize the Glisson capsule, which is known to evoke phrenic pain [72]. However, most patients do not relate their NP and the concomitant visceral disorder, or fail to report gastrointestinal or hepatic/biliary symptoms [15], which contributes to the misdiagnosis of NP as mechanical or non-specific.

It is interesting to note that experimental research of gastric sensitivity is performed in rats by means of gastric distension, which is very common in obesity, and is related to dyspepsia. This gastric distension triggers an increase in the electromyographic activity of the neck muscles and also affects to the neck posture [89]. The addition of substances which increase the insult to the stomach enhances this visceromotor response [84]. The same mechanisms have been used to experimentally study the gastric hypersensitivity frequently observed in patients with long-standing diabetes [90]. The increase of muscle tone in the area of referred hyperalgesia does not appear only when the stomach is injured, since it has also been demonstrated by artificial ureteric stones [91]. Moreover, the neck muscles’ tone decreased in these models when the viscera was treated by means of electrical stimulation [92,93]. Likewise, manual visceral treatment has also been shown to improve NP and electromyographic recordings of the upper trapezius muscles of subjects suffering from chronic non-specific NP and dyspepsia [94], and has improved neck mobility and NP thresholds in subjects suffering from gastroesophageal reflux disease [95]. On the contrary, the likely participation of patients with NP of visceral origin might contribute to explaining the scarce success of usual treatments for NP, achieving at most moderate effects in the short-term [96].

Therefore, the visceral origin of NP might be more easily diagnosed if it only triggers VRP, because there will be no modification of pain related to activity or posture. However, as previously exposed, mechanical consequences can be also elicited in case of visceral aetiology of NP [15,24,61,63,64,66,84,89,90,91,92,93], hindering the correct diagnosis. The presence of muscle hypertonus, myofascial trigger points, and/or myofascial pain syndrome may increase symptoms during musculoskeletal activity. Similarly, pain modification related to movement and/or postures has also been described during the affectation of the spleen [97], gallbladder [72,98], kidney [99,100], and heart [15,101].

### 4.3. Needs for the Future

Our results suggest that clinical trials about NP fail to suspect a visceral origin of NP. This poor consideration seems to be shared in the research and clinical settings, considering that authors from clinical institutions participated in nearly 80% of the studies reviewed. Thus, our study points out the need to further develop the knowledge of somatic consequences of visceral disorders, at least when related to NP. Additionally, it shows the need for more research to get to know the prevalence of NP of visceral origin in different settings (primary care, clinics of rehabilitation, chiropractic, osteopathy, and physiotherapy).

### 4.4. Limitations

The present findings should be carefully interpreted for several reasons. First, the search strategy was conducted in a single database. The aim was to select those trials with higher impacts, based on the number of citations or on the publication in first quartile journals of the Journal Citation Reports. This strategy was set in order to select those trials which could be representative of the best research about neck pain; i.e., the most cited (showing that they are used as a reference by many researchers) and those published in the best quality journals (which are supposed to publish the best studies). The Web of Science database provides the number of citations and the quartile, and it is considered of high prestige in the Health Sciences field. Other databases such as Medline do not provide numbers of citations nor journal impact factors. Other reviews used different strategies to show a general overview about a subject, such as random selection of studies and/or selection of specific major journals [102,103,104,105]. Second, with respect to sample size, it could be argued that our sample size (30 studies) is not big enough to be representative. The right sample size to perform this kind of study has not been established. In the literature, systematic reviews about research bias can be found with sample sizes ranging between 10 and 44 studies [106,107,108,109,110,111]. Third, despite the high prevalence of visceral disorders, it is not possible to conclude that these studies actually included patients with VRP to the neck area. Therefore, the influence of this issue on the results of each trial remains unknown.

## 5. Conclusions

In conclusion, it seems that most of the top cited and most recent clinical trials assessing treatments in NP lacked the consideration of visceral referred NP according to their eligibility criteria, showing that neck pain of visceral origin is underestimated. Although NP referred from viscera is difficult to diagnose, more stringent inclusion and exclusion criteria may be required in clinical trials. Otherwise, this may imply an incorrect estimation of the usefulness of the interventions.

## Figures and Tables

**Table 1:** Top 15 most cited clinical trials about treatment efficacy in neck pain published between 1995 and 2018. NP, neck pain; Clinical, number of authors belonging to clinical institutions; Non-Clinical, number of authors belonging to academic institutions; SM, spinal manipulation; PT, physical therapy; GP, general practitioner.

Study Authors’ Institutions Number of Citations	PEDro Score	Aim	Participants (Sex and Mean Age)	Inclusion Criteria	Exclusion Criteria
Ylinen et al., 2003 [32] Clinical: 5 Non-Clinical: 4 Citations: 285	7/10	Assess the efficacy of intensive isometric training and light endurance training in chronic NP	N = 180 All females 46 years	Females; aged 25 to 53 years; office worker; permanently employed; motivated to continue working and for rehabilitation; constant or frequently occurring NP > than 6 months	Severe neck disorders, e.g., disk prolapse and spinal stenosis; postoperative conditions in the neck-shoulder; severe trauma; instability; spasmodic torticollis; frequent migraine; peripheral nerve entrapment; fibromyalgia; shoulder tendonitis, bursitis, or capsulitis; inflammatory rheumatic disease; severe psychiatric illness; diseases that prevent physical loading; pregnancy
Cleland et al., 2005 [40] Clinical: 5 Non-Clinical: 0 Citations: 156	8/10	Evaluate the immediate effects of thoracic SM in chronic NP	N = 36 27 females 9 males 35 years	Aged 18 to 60 years; primary complaint of mechanical NP, defined as non-specific pain in the cervicothoracic region and exacerbated by neck movements	Red flags for serious spinal conditions, e.g., infection, tumors, osteoporosis, fracture; positive signs or symptoms suggestive of nerve root involvement, e.g., altered upper limb reflexes, sensation, or strength; cervical or thoracic surgery; prior SM treatment; thoracic spine hypermobility; pregnancy
Jordan et al., 1998 [36] Clinical: 3 Non-Clinical: 3 Citations: 152	5/10	Assess the effectiveness of intensive cervical training vs. PT vs. chiropractic treatment in chronic NP	N = 119 88 females 31 males 39 years	Aged 20 to 60 years; NP > 3 months with or without non-radicular pain; to live within a close distance to the hospital; X-ray examination of the cervical spine; to be able to speak and read Danish	Acute NP with no freedom of movement; PT, SM, or training for the neck-upper extremity within 6 months; neuropathy; systemic disease; inflammatory joint or muscle disease; headache dominating over NP; migraine; hypermobility; whiplash; primary shoulder or upper extremity problems; previous neck surgery
Irnich et al., 2001 [41] Clinical: 0 Non-Clinical: 10 Citations: 146	7/10	Compare the efficacy of acupuncture vs. massage combined with “sham” laser acupuncture in chronic NP	N = 177 117 females 60 males 52 years	Aged 18 to 85 years; chronic NP; painful restriction of neck mobility > 1 month; had not received any treatment in the previous 2 weeks	Previous surgery, dislocation or fracture; neurological deficits; systemic disorders; contraindications to any of the applied treatments
Korthals-de Bos et al., 2003 [42] Clinical: 0 Non-Clinical: 9 Citations: 137	6/10	Evaluate the cost effectiveness of PT, manual therapy, and GP care for acute, subacute and chronic NP	N = 183 121 females 62 males 45 years	Aged 18 to 70 years; NP > 2 weeks (confirmed during physical examination); willingness to comply with treatment and follow up	PT or manual therapy for NP in the previous 6 months; neck surgery; a specific cause for the NP (for example, malignancy, fracture, or inflammation)
Cleland et al., 2007 [43] Clinical: 4 Non-Clinical: 2 Citations: 133	7/10	Compare the effect of thoracic nonthrust vs. mobilization/SM, and compare frequencies, side effects, and durations in acute and subacute NP	N = 60 33 females 27 males 43 years	Aged 18 to 60 years; a primary complaint of NP with or without unilateral upper-extremity symptoms; a baseline Neck Disability Index score ≥ 10%	Signs suggestive of a non-musculoskeletal aetiology; whiplash within 6 weeks; cervical spinal stenosis; signs of nerve root compression (decrease of at least 2 of the following: myotomal strength, sensation, or reflexes); central nervous system involvement; previous cervicothoracic surgery; pending legal action
Irnich et al., 2002 [44] Clinical: 0 Non-Clinical: 8 Citations: 127	6/10	Evaluate immediate effects of 2 different modes of acupuncture vs. sham procedure in chronic NP	N = 34 25 females 9 males 52 years	NP > 2 months; diagnosis of myofascial syndrome or irritation syndrome based on history, pain characteristics, radiological findings and manual examination	Radicular cervical syndrome; segmental instability; fracture or surgery of the cervical spine; contradictions to acupuncture; drug, PT or manual treatment in the last 4 weeks
Viljanen et al., 2003 [31] Clinical: 6 Non-Clinical: 0 Citations: 117	8/10	Assess the effectiveness of dynamic muscle training and relaxation training in chronic NP	N = 393 All females 45 years	Female sex; aged 30 to 60 years; suffer from chronic non-specific NP > 12 weeks	Cancer; major trauma; rheumatic disease; neural entrapment; major rehabilitation in the previous 3 months
Hurwitz et al., 2002 [45] Clinical: 0 Non-Clinical: 6 Citations: 112	7/10	Compare the relative effectiveness of cervical SM and mobilization in acute, subacute and chronic NP	N = 336 231 females 105 males 46 years	Aged 18 to 70 years; NP, defined as pain within the upper thoracic spine to the occiput and the surrounding musculature; members of health maintenance organization; had sought care at one of the study sites; had not received NP treatment in the past month	NP due to fracture, severe spondyloarthropathy, tumor, infection, or other non-mechanical cause; progressive neurological deficit, myelopathy, herniated nucleus pulposus, or severe incapacitating pain; severe coexisting disease; previous electrotherapy treatment; blood coagulation disorder; use of anticoagulant or corticosteroids; stroke or transient ischemic attacks; inability to read English; pain involving third-party liability or compensation
White et al., 2004 [46] Clinical: 2 Non-Clinical: 2 Citations: 104	7/10	Compare acupuncture and placebo in chronic NP	N = 135 87 females 48 males 53 years	Aged 18 to 80 years; mechanical NP > 2 months; pain score > 30 mm on a Visual Analogue Scale for 5 of 7 days before treatment	Previous neck fracture or surgery; cervical congenital abnormality; uncontrolled low back pain; contraindication to acetaminophen; systemic illness, e.g., rheumatoid arthritis; ongoing litigation or disability claims; current or recent manual neck treatment or steroid use (oral or local injection); or pregnancy
Evans et al., 2002 [47] Clinical: 1 Non-Clinical: 3 Citations: 102	7/10	Compare the effects of SM combined with low-tech rehabilitative exercise, MedX rehabilitative exercise, or SM alone in chronic NP	N = 191 113 females 78 males 44 years	Aged 20 to 65 years; mechanical NP > 12 weeks; no specific, identifiable aetiology (i.e., inflammatory disease, infection); pain reproduced by neck movement or provocation tests and localized between the most inferior part of the occipital bone and T1 spinous process	NP referred from peripheral joints or viscera; progressive neurologic deficits; severe osteopenia; vascular disease of the neck or upper extremity; significant infectious disease or other severe disabling health conditions; previous neck surgery; inability to work because of NP; current or pending litigation, SM or exercise therapy within 3 months; concurrent treatment for NP by other health care providers
Manchikanti et al., 2010 [38] Clinical: 3 Non-Clinical: 1 Citations: 96	10/10	Evaluate the clinical outcomes of therapeutic cervical medial branch blocks with local anesthetic with or without steroids in chronic NP of facet joint origin	N = 120 89 females 31 males 45 years	Function-limiting NP > 6 months; 18 years or older; to provide written informed consent; positive results with controlled diagnostic cervical facet joint nerve blocks (80% pain relief and the ability to perform previously painful movements)	Disc herniation with radicular pain; symptomatic spinal stenosis; neck surgery within 3 months; uncontrolled major depression or psychiatric disorders; heavy opioid usage; acute or uncontrolled medical illness; chronic severe conditions; inability to stay in a prone position; history of adverse reactions to local anesthetics or steroids; or pregnant or lactating women
Hoving et al., 2006 [48] Clinical: 0 Non-Clinical: 11 Citations: 92	8/10	Compare the effectiveness of manual therapy, PT and continued care by the GP over a 1 year period	N = 183 111 females 72 males 45 years	Aged 18 to 70 years; pain and/or stiffness in the neck > 2 weeks; nonspecific neck complaints reproducible during active or passive range of motion; willingness to participate	No specific cause for the pain, e.g., systemic disease, fracture, or organic disorders; a history of trauma or additional dominant complaints, such as headache or nonradicular pain; previous neck surgery; manual or physiotherapy in the previous 6 months
Chiu et al., 2005 [49] Clinical: 0 Non-clinical: 3 Citations: 86	7/10	Evaluate the efficacy of a neck exercise program in chronic NP	N = 145 100 females 45 males 44 years	Aged 20 to 70 years; NP (of various intensity of pain) > 3 months; able to read Chinese	Previous neck or upper back (T1-T6) injury; inflammatory condition, e.g., rheumatoid arthritis; former neck surgery; a malignancy or congenital spinal abnormality; parallel NP treatment; contraindication for infrared irradiation; neurologic symptoms, e.g., muscle weakness or changes in spinal reflex jerks; other musculoskeletal problems; acute NP with no freedom of movement; training or SM for NP within 6 months; work-related injuries
Bronfort et al., 2012 [50] Clinical: 3 Non-clinical: 3 Citations: 81	7/10	Determine the relative efficacy of SM, medication, and home exercise with advice for acute and subacute NP in the short and long term.	N = 272 178 females 94 males 48 years	Aged 18 to 65 years; primary symptom of mechanical, nonspecific NP equivalent to grades I or II of the Bone and Joint Decade 2000–2010 Task Force on NP and Its Associated Disorders classification; NP between 2–12 weeks duration; NP ≥ 3 on a 0 to 10 scale; not seeking additional NP treatment	Cervical spine instability; fracture; NP referred from peripheral joints or viscera; progressive neurologic deficits: cardiac disease requiring medical treatment; blood clotting disorders; diffuse idiopathic hyperostosis; inflammatory or destructive tissue changes of the cervical spine; infectious disease; substance abuse; cervical spine surgery; severe disabling health problems; pending or current litigation; having received any of the study treatments within 3 months; pregnancy or breastfeeding

**Table 2:** List of the fifteen most recent clinical trials, by November 2018, about treatment efficacy in neck pain published in high impact journals. NP, neck pain; Clinical, number of authors belonging to clinical institutions; Non-Clinical, number of authors belonging to academic institutions; NDI, neck disability index; PT, physical therapy; VAS, visual analogue scale; SM, spinal manipulation; N/S, non-clearly specified.

Study Authors’ Institutions Number of Citations	PEDro Score	Aim	Participants	Inclusion Criteria	Exclusion Criteria
Celenay et al., 2016 [51] Clinical: 0 Non-Clinical: 3 Citations: 13	6/10	Assess the effect of neck stabilization and scapulo-thoracic treatment with and without connective tissue massage in chronic NP	N = 60 39 females 21 males 48 years	Aged 18 to 65 years; NP > 3 months; baseline NDI ≥ 20%	Stenosis; traumatic injury history; previous neck surgery; cancer; hypermobility; inflammatory rheumatologic diseases; severe psychological disorders; exercise or PT intervention in the last 3 months; pregnancy
Celenay et al., 2016 [52] Clinical: 0 Non-Clinical: 3 Citations: 8	7/10	Compare the effect of stabilization exercises with or without manual therapy in patients with mechanical chronic NP	N = 102 74 females 28 males 46 years	Aged 18 to 65 years; NP > 3 months, with symptoms provoked by postures, movements, or palpation	Inflammatory rheumatologic diseases, structural deformity, or malignity; previous cervical surgery; spinal stenosis; bilateral upper extremity symptoms; ≥ 2 positive radicular signs of nerve root compression; referred pain > than 7 on a 0-10 VAS in the related dermatome in the upper extremities; capsular pattern of arthritis; severe psychological disorder; pregnancy; any intervention including exercise or PT within 3 months
Cerezo et al., 2016 [30] Clinical: 4 Non-Clinical: 4 Citations: 10	6/10	Assess the effect of deep dry needling of myofascial trigger points in chronic nonspecific NP	N = 128 Sex distribution: N/S 50 years	NP (with or without radiation) > 6 months, with no known pathological basis (neurological, trauma); having myofascial pain syndrome	Major trauma; widespread pain; inflammatory, hormonal, or neurological disorders; upper limbs tendinopathy; severe psychiatric illness; inability to speak or write Spanish; use of muscle relaxant, analgesic, antidepressant, or anticoagulant medication in the last week; fibromyalgia; any contraindication to PT (infection, fever, hypothyroidism, wounds, metal allergy, cancer or systemic disease, fear of needles); or pregnancy
De Araujo et al., 2018 [34] Clinical: 0 Non-Clinical: 5 Citations: 0	8/10	Assess the effectiveness of the Pilates method in chronic NP	N = 64 14 females 50 males 49 years	Aged 18 to 65 years; non-specific NP according to the Neck Pain Task Force; pain > 3 months; and pain intensity between 3–8 cm on a 0 to 10 cm rating scale	Fibromyalgia; spine trauma, infection or inflammation; NP radiating to the upper limbs; having started or changed physical activity > 2/week within 3 months; visual impairments and no use of glasses; new or changed pain medication, or injections in the last 3 months; neurological diseases; musculoskeletal diseases hindering the practice of Pilates; pregnancy
Essex et al., 2017 [37] Clinical: 1 Non-Clinical: 16 Citations: 0	4/10	Assess the cost-effectiveness of usual care vs. acupuncture and usual care vs. Alexander Technique and usual care for chronic NP	N = 517 347 females 170 males 53 years	NP > 3 months; score > 28% on the Northwick Park Neck Pain Questionnaire	Current acupuncture treatment for NP or attended Alexander lessons in the last 2 years; litigation; serious underlying pathology; prior neck surgery; alcohol or drug dependency; involvement in other trial; history of psychosis, rheumatoid arthritis, osteoporosis, hemophilia, ankylosing spondylitis, cancer, HIV or hepatitis; inability to speak English; pregnancy
Fernández-Carnero et al., 2018 [35] Clinical: 3 Non-Clinical: 2 Citations: 0	8/10	Assess the immediate effect of neural tension technique in chronic NP	N = 54 41 females 13 males 21 years	Aged 18 to 65 years; NP within the nuchal line and T1 spinous process > 12 weeks; no radicular symptoms to head, trunk, or upper limbs; ability to write and speak Spanish	Systemic or degenerative diseases; headache and/or low back pain within 9 months; NP linked with whiplash; moderate or severe depression; red flags (metabolic diseases, tumor, fracture, rheumatoid arthritis, osteoporosis); fibromyalgia; neck surgery; cervical radiculopathy; disc herniation; neck or face pain within 6 months; NP with vertigo caused by vertebrobasilar insufficiency; non-cervicogenic headache after trauma within last year
Griswold et al., 2018 [53] Clinical: 1 Non-Clinical: 4 Citations: 0	7/10	Compare the effect of concordant cervical and thoracic non-thrust vs. thrust SM for chronic mechanical NP	N = 103 76 females 27 males 47 years	Aged 18 to 70 years; having mechanical NP; NDI ≥ 20%; and NP > 2 on a 0 to 10 rating scale in the last 24 h	Contraindications to manual therapy (fracture, malignancy, rheumatoid arthritis, myelopathy, osteoporosis); prior cervical or thoracic spine surgery; seeking litigation; nerve root compression (at least 2 or more neurological signs); receiving other nonsurgical care; inability to reproduce the concordant sign in the cervical or thoracic spine during testing
Krøll et al., 2018 [54] Clinical: 4 Non-Clinical: 1 Citations: 1	5/10	Evaluate the efficacy of aerobic exercise in migraine and coexisting tension-type headache and chronic NP	N = 70 62 females 8 males 37 years	A minimum of 2 attacks of migraine; a minimum of 1 day with tension-type headache; a minimum of 1 day with NP per month	Whiplash; significant neck trauma, (fracture, distortion, or violent attack); neck nerve root compression; persistent headache linked with trauma; medication overuse; severe physical and/or mental illness; trigeminal neuralgia; cluster headache; alcohol and drugs abuse; breastfeeding; inability to speak Danish; pregnancy
Lauche et al., 2016 [55] Clinical: 1 Non-Clinical: 8 Citations: 7	7/10	Evaluate the efficacy of Tai Chi for treating chronic NP	N = 114 91 females 23 males 49 years	Age > 18 years; nonspecific NP > 3 consecutive months for at least 5 days a week; NP > 45 mm on a 0 to 100 mm VAS	NP caused by trauma, disc protrusion, whiplash, spinal deformity, stenosis, neoplasm, neurological disorder, rheumatic or active severe affective disorder, addiction, psychosis, or oncologic disease; invasive spinal treatment within 4 weeks; spinal surgery in the last year; new or modified drug regimen; opioids intake; regular practice of Tai Chi, Qigong, or Yoga within 6 months; any disability precluding exercise; pregnancy
Lauche et al., 2016 [56] Clinical: 3 Non-Clinical: 5 Citations: 3	6/10	Assess the efficacy of the Alexander Technique, local heat and guided imagery in patients with chronic non-specific NP	N = 72 65 females 7 males 41 years	Aged 18 to 50 years; non-specific NP > 3 months; NP intensity > 40 mm on a 100 mm VAS	NP caused by disc protrusion or prolapse; spinal congenital deformity; spinal stenosis; whiplash; neoplasm, inflammatory rheumatic disease; neurological disorder; active oncologic disease; affective disorder; addiction; psychosis; previous spinal surgery or invasive spinal treatment within 3 weeks; ongoing application for disability pension; previous Alexander technique experience; participation in other clinical trials; pregnancy
Monticone et al., 2017 [57] Clinical: 6 Non-Clinical: 1 Citations: 3	8/10	Evaluate the effect of a group based multidisciplinary rehabilitation programme in chronic NP	N = 170 121 females 49 males 53 years	Age >18 years; documented history of non-specific NP >3 months; a good understanding of Italian	Acute and subacute NP; cognitive impairment; clear aetiology for their NP, e.g., previous spinal surgery, deformity, disc herniation, infection, fracture, myelopathy or malignancy, whiplash, and systemic or neuromuscular diseases; having received cognitive-behavioral therapy
Pillastrini et al., 2016 [58] Clinical: 1 Non-Clinical: 7 Citations: 6	8/10	Evaluate the effectiveness of global postural reeducation vs. manual therapy in chronic NP	N = 96 72 females 22 males 48 years	Nonspecific NP > 3 months; aged 18 to 80 years; ability to read and speak Italian	Acute or subacute NP; specific cause of NP, e.g., systemic, rheumatic, neuromuscular diseases; central or peripheral neurological signs; cognitive impairment, spinal surgery; or PT treatments in the prior 6 months
Ris et al., 2016 [59] Clinical: 1 Non-Clinical: 5 Citations: 7	6/10	Assess the effect of pain education, exercises and graded physical activity vs. pain education alone in chronic NP	N = 200 149 females 51 males 45 years	Aged >18 years; traumatic or non-traumatic NP > half a year; NDI >10; NP, primary pain; complete medical diagnostic procedures	Clinically confirmed radiculopathies; progressive medical treatment; unstable social/working conditions; current fractures; score > 29 in the Beck Depression Inventory-II; conditions limiting participation; pregnancy
Thompson et al., 2016 [33] Clinical: 2 Non-Clinical: 1 Citations: 4	5/10	Evaluate the effect of physiotherapist led cognitive—behavioral intervention plus exercise in chronic NP	N = 57 27 females 28 males 48 years	Non-specific NP > 3 months; fluency in English; have not received PT for NP in the past 3 months	Serious pathology (fracture, dislocation, carcinoma or infection); radiculopathy; myelopathy; rheumatological disorder; diagnosed major psychiatric illness
Tunwattanapong et al., 2016 [60] Clinical: 1 Non-clinical: 2 Citations: 10	8/10	Determine the effect of neck and shoulder stretching exercises for chronic NP among office workers	N = 96 87 females 9 males 35 years	Office workers who rated themselves with moderate to severe neck or shoulder pain (VAS ≥ 5 of 10 cm) for more than 3 months	Performing regular stretching exercise; a history of severe neck injury, or neck or shoulder contracture (defined by a limitation range of motion in all directions); previous neck or shoulder surgery; abnormal neurological signs
